# Supplementary material for: Surface hydrophobization of hydrogels via interface dynamics-induced network reconfiguration
Source: Nat Commun. 2024 Jan 3;15:239. doi: 10.1038/s41467-023-44646-5 (PMC10764767; doi:10.1038/s41467-023-44646-5)
Supplement: Supplementary file 1 — Supplementary Information [file 41467_2023_44646_MOESM1_ESM.pdf]

Supplementary Information

Surface hydrophobization of hydrogels via interface dynamics-induced network reconfiguration

Bo Yi *et al.*

Corresponding author: Yi Wang, yiwang@cuhk.edu.hk; Zuankai Wang, zk.wang@polyu.edu.hk; Liming Bian, bianlm@scut.edu.cn

This PDF file includes:

Supplementary Discussion 1–2
Supplementary Figures 1–34

Supplementary Discussion

1. Comparison of silicone oil-coated mold and DNR mold for hydrogel preparation

Silicone oil-coated mold and DNR mold were used to prepare PAA hydrogels to demonstrate the importance of covalently grafting silicone chains onto the mold surface for hydrogel surface wettability regulation (Supplementary Fig. 8a). Before hydrogel preparation, both the silicone oil-coated mold and DNR mold showed hydrophobicity (Supplementary Fig. 8b). However, the WCA value of the hydrogel prepared by the oil-coated mold was much lower and declined much faster than that of the DNR hydrogel (Supplementary Fig. 8c and 8d). Moreover, the silicone oil was entrained by the hydrogel upon extraction from the mold, as confirmed by the emerging signal of siloxane in the ATR-FTIR curve (Supplementary Fig. 8e). This resulted in a dramatic decrease in the WCA of the oil-coated mold after hydrogel preparation (Supplementary Fig. 8b). In contrast, the WCA of the DNR mold did not change even after 20 uses for hydrogel preparation (Supplementary Fig. 8f).

2. Determination of the grafting density of silicone chains on the DNR mold surface

We determined the density of silicone chains on the DNR mold surface by AFM topographical imaging to quantify the fraction of the mold surface occupied by silicone based on the color difference (Supplementary Fig. 20). To make the color difference more observable and quantifiable, we converted the original AFM images to gray mode and split the color channel to red with a threshold of 80 by ImageJ. The red region represents the mold substrate, while the gray region is the silicone. The fraction of silicone chains was determined by measurement of the proportion of the gray region (Fig. 2g).

Supplementary Figures

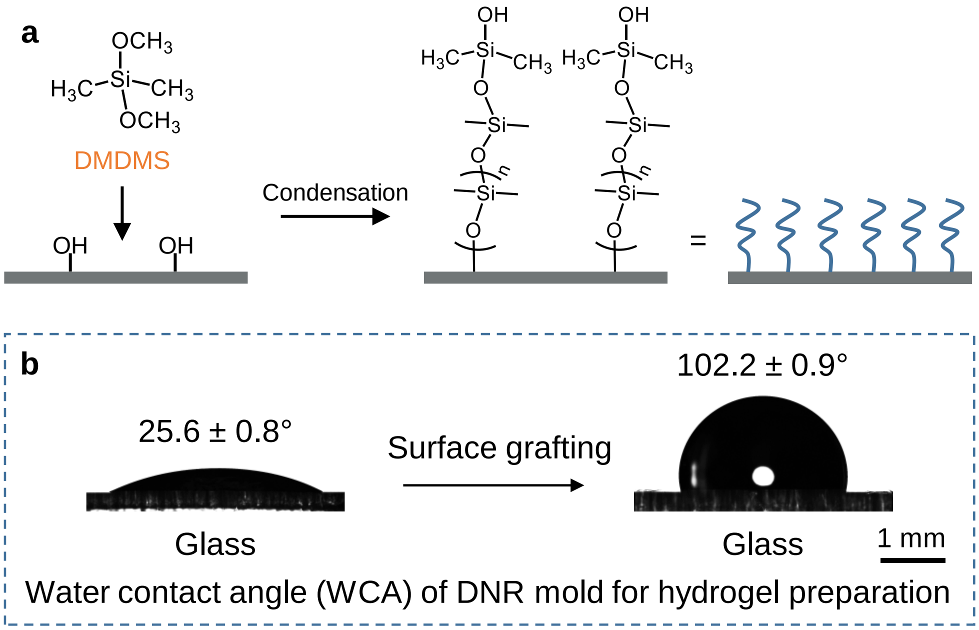

Supplementary Fig. 1 | Silicone chain grafting on molds. a Silicone chains were grafted onto hydroxyl-bearing molds via the hydrolysis and condensation reactions of dimethyldimethoxysilane (DMDMS). **b** The glass mold shows a transition of wettability (hydrophilic to hydrophobic) after silicone chain grafting.

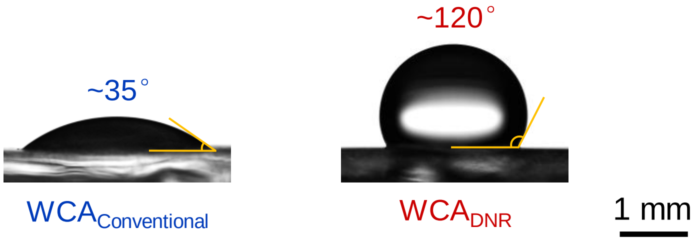

Supplementary Fig. 2 | Water contact angles (WCAs) of conventional and DNR hydrogels. The poly(acrylic acid) (PAA) hydrogels were prepared using the conventional method or the DNR strategy.

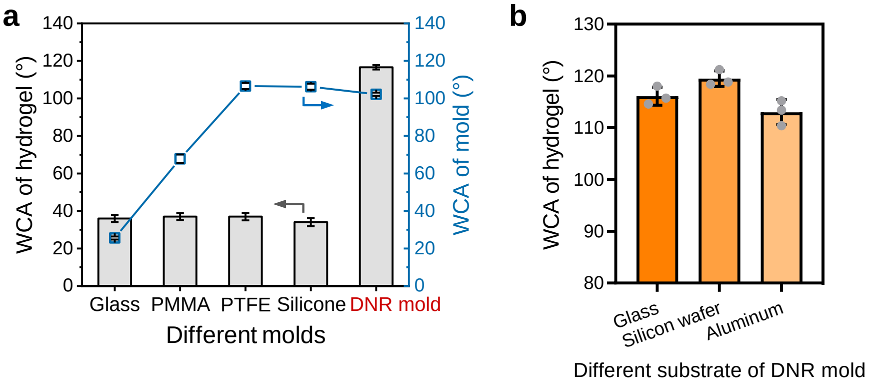

Supplementary Fig. 3 | Surface wettability of PAA hydrogels prepared by different molds. a WCA of different mold substrates (blue curve) and corresponding WCA of PAA hydrogels prepared using the molds (gray columns). The substrate of the DNR mold is glass. **b** WCA of hydrogels prepared by the DNR mold with different substrate materials. Notably, because PMMA and PTFE are chemically inert and the surface modification of the two substrates with organosilanes is highly challenging, we did not prepare DNR molds by PMMA and PTFE substrates. Data are shown as the mean ± SD; n = 3 independent samples.

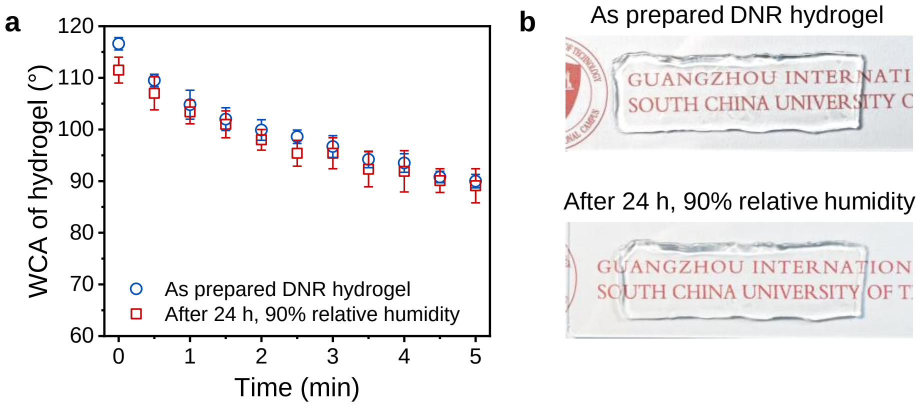

Supplementary Fig. 4 | a WCA evolution and **b** photo images of the PAA-based DNR hydrogel after preparation and standing for 24 h at room temperature and 90% relative humidity. Values in **a** are shown as the mean ± SD; n = 4 independent samples.

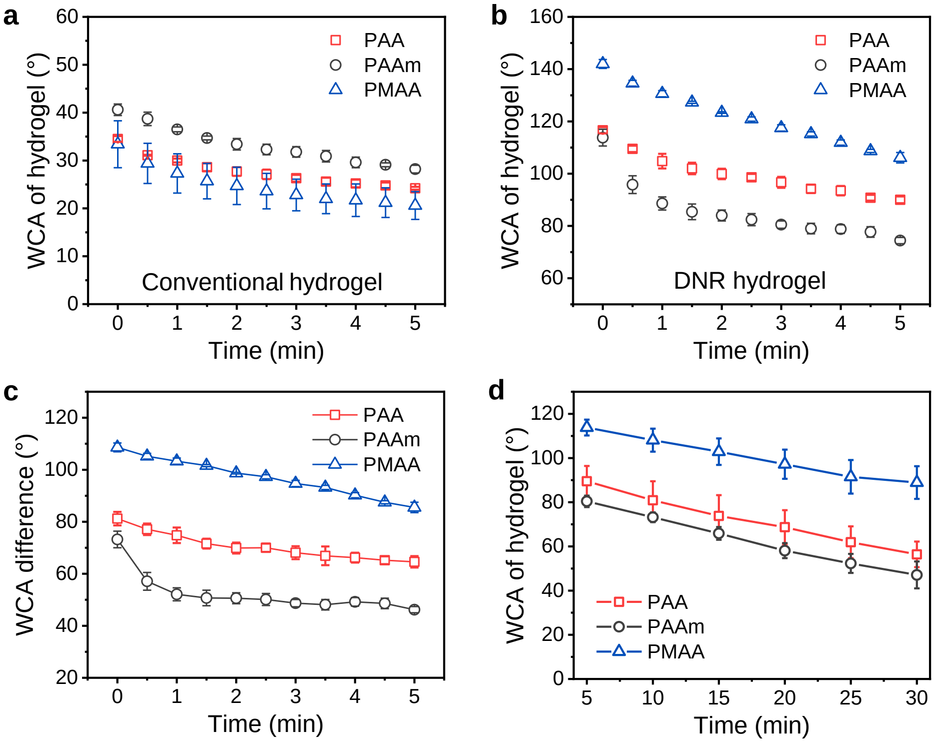

Supplementary Fig. 5 | Surface wettability of different hydrogels prepared by the conventional method or DNR strategy. a WCA evolution of three kinds of conventional hydrogels (PMAA, PAA and PAAm hydrogels) in 5 min. **b** WCA evolution of the corresponding DNR hydrogels in 5 min. **c** WCA difference of the above three hydrogels. WCA difference means the WCA value of the DNR hydrogel minus that of the conventional hydrogel. **d** WCA evolution of the PAA-based DNR hydrogel in 30 min. Data are shown as the mean ± SD; n = 4 independent samples.

Discussion: The more significant reduction of WCA for PAAm hydrogel is possibly due to the lower polymer content of PAAm hydrogel (20 wt%) compared to that (25 wt%) of PAA and PMAA hydrogels, which like makes the hydrogel more permeable by the probing water droplet. On the other hand, we reason that the difference in side functional groups, i.e., amino in PAAm and carboxyl in PAA/PMAA, could also result in the varied recovery rate of the hydrogel surface network when contacting with the probing water droplet.

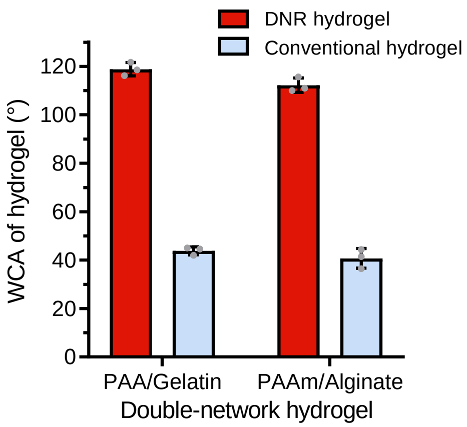

Supplementary Fig. 6 | WCA of double-network hydrogels prepared by the conventional method or DNR strategy. Data are shown as the mean ± SD; n = 3 independent samples.

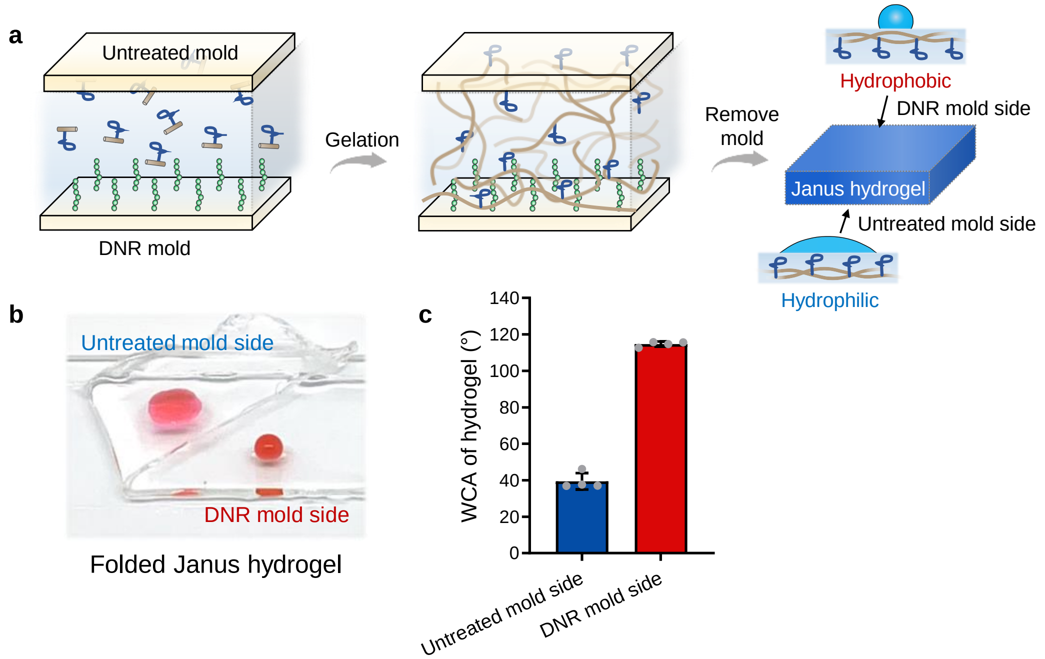

Supplementary Fig. 7 | Fabrication of the Janus hydrogel with asymmetric wettability using the DNR strategy. a Schematic illustration of the fabrication process by placing the untreated mold and DNR mold on the different sides of the hydrogel. **b** Image shows the Janus hydrogel with totally different wettability on the two sides of the hydrogel. Water droplets were dyed by rhodamine to improve visibility. **c** Comparison of the WCA on the two sides of the Janus hydrogel. Values in **c** are shown as the mean ± SD; n = 4 independent samples.

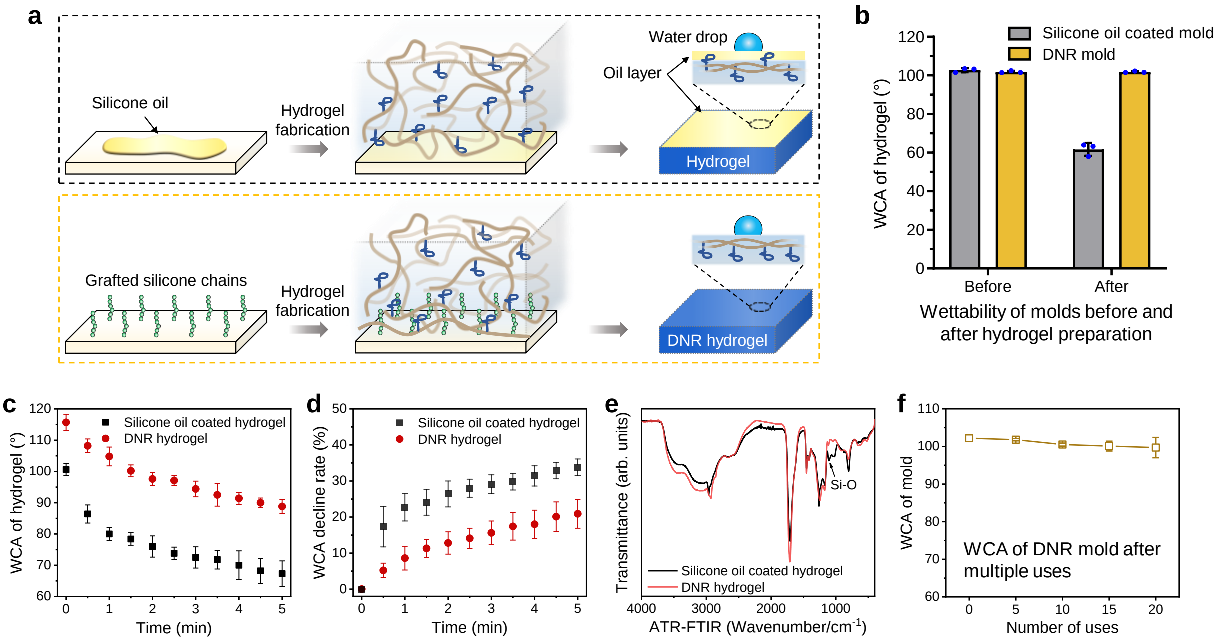

Supplementary Fig. 8 | Comparing silicone oil-coated mold and DNR mold for hydrogel surface wettability regulation. **a** Schematic of the hydrogel preparation process by using a silicone oil-coated mold or DNR mold. **b** WCA of the molds before and after hydrogel preparation. **c** WCA evolution, **d** Corresponding decline percentage of WCA, and **e** ATR-FTIR spectra of the PAA hydrogels prepared by silicone oil-coated mold (named silicone oil-coated hydrogel) and DNR mold. **f** WCA of the DNR mold after 20 uses for hydrogel preparation. Values in **b–d** and **f** are shown as the mean ± SD; n = 3 independent samples.

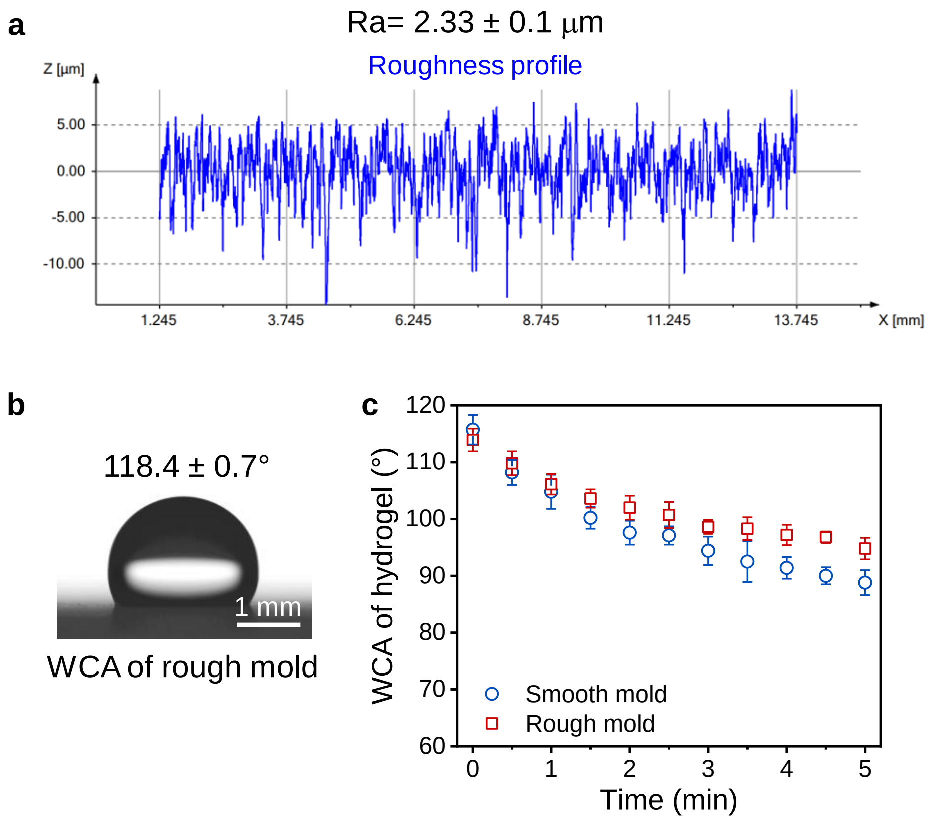

Supplementary Fig. 9 | Influence of mold surface roughness on hydrogel surface wettability. **a** Average roughness (Ra) of a laser-polished glass mold measured by a roughmeter (W912C, Jenoptik). **b** WCA of a rough DNR mold, which shows higher hydrophobicity than a smooth DNR mold. **c** WCA evolution of hydrogels prepared by smooth or rough DNR molds. Values in **c** are shown as the mean ± SD; n = 4 independent samples.

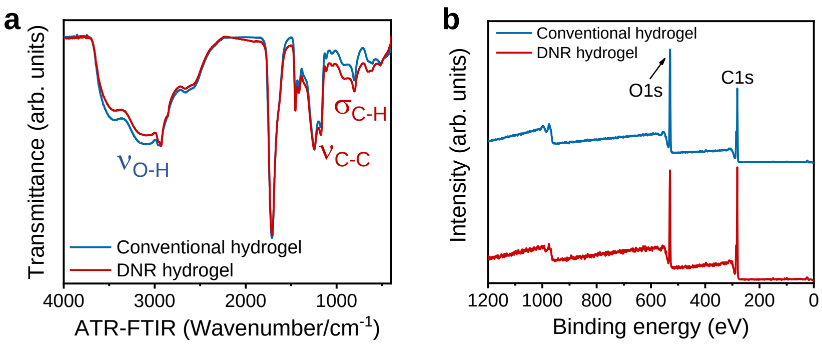

Supplementary Fig. 10 | a ATR-FTIR and **b** *XPS* spectra of conventional and DNR hydrogels. To make the contrast of *XPS* curves more distinctive, the overall intensity of DNR hydrogel was adjusted to being comparable with the conventional hydrogel.

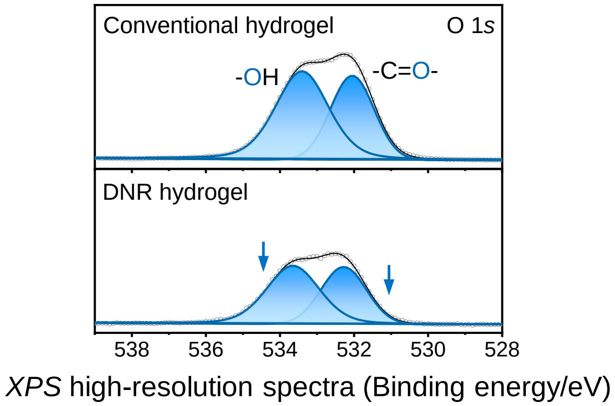

Supplementary Fig. 11 | High-resolution *XPS* O 1*s* spectra with deconvolution of polymer structures for conventional and DNR hydrogels.

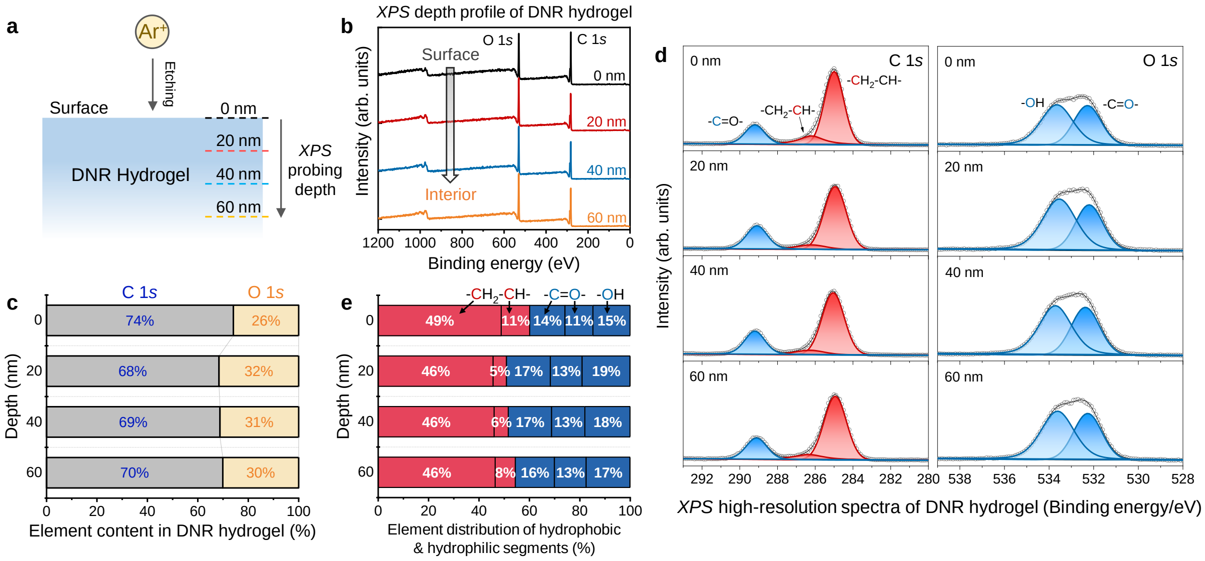

Supplementary Fig. 12 | a Schematic of surface etching for *XPS* profiling of the DNR hydrogel surface at various depths. **b** *XPS* depth profile of the DNR hydrogel with depth from the top surface to ~60 nm. **c** Element contents of carbon and oxygen on the DNR hydrogel surface at various depths. The values were determined by integration of the C 1*s* and O 1*s* peak areas. **d** High-resolution *XPS* spectra of the DNR hydrogel depth profile. **e** Statistics of the element content distribution of polymer segments in the DNR hydrogel at various depths.

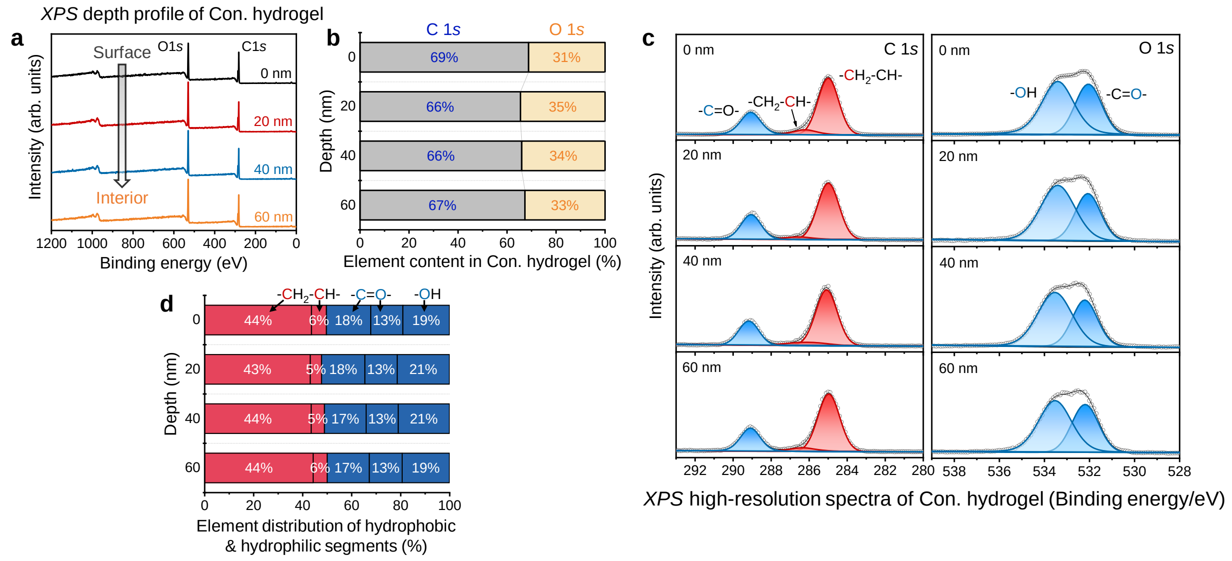

Supplementary Fig. 13 | a *XPS* depth profile of the conventional (Con.) hydrogel with depth from the top surface to ~60 nm. **b** Element contents of carbon and oxygen on the conventional hydrogel surface at various depths. The values were determined by integration of the C 1*s* and O 1*s* peak areas. **c** High-resolution *XPS* spectra of the conventional hydrogel depth profile. **d** Statistics of the element content distribution of polymer segments in the conventional hydrogel at various depths.

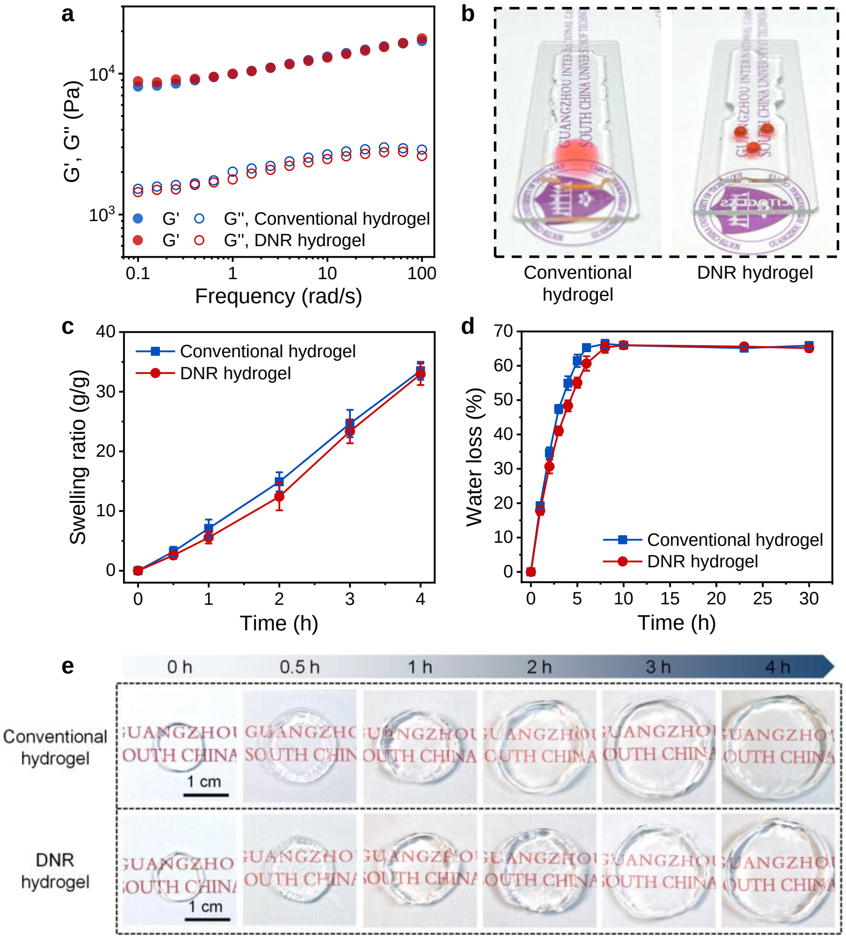

Supplementary Fig. 14 | Bulk properties of conventional and DNR hydrogels. a Rheological frequency sweep, **b** Images showing transparency, **c** swelling ratio, and **d** water loss ratio of conventional and DNR hydrogels. Water droplets dyed by rhodamine were dropped on hydrogel surfaces to distinguish DNR hydrogel from conventional hydrogel and improve visibility. **e** Photo images of conventional and DNR hydrogels over time during the swelling test. Values in **c** and **d** are shown as the mean ± SD; n = 3 independent samples.

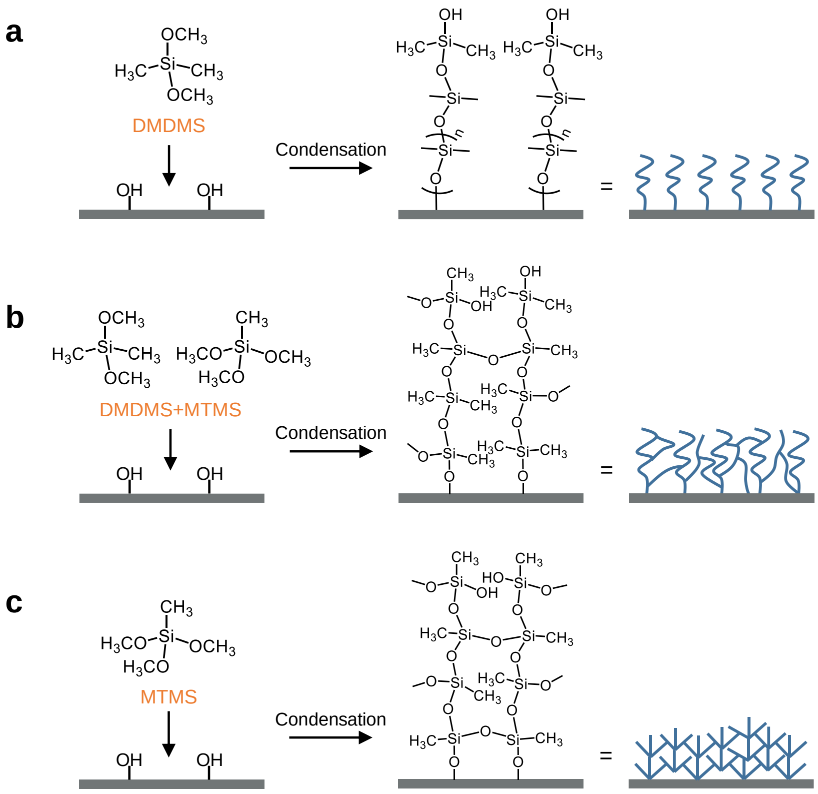

Supplementary Fig. 15 | Regulation of the crosslinking degree of silicone chains using methyltrimethoxysilane (MTMS). The proportion of MTMS to DMDMS in the reactive silane solution is **a** 0 wt% MTMS, **b** 50 wt% MTMS, and **c** 100 wt%.

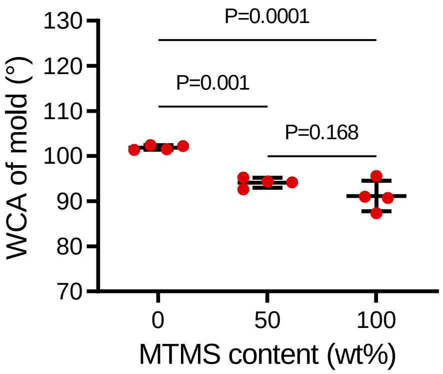

Supplementary Fig. 16 | WCA of the DNR molds prepared with different crosslinking degrees of silicone chain, which were regulated by the MTMS content. Data are shown as the mean ± SD; n = 4 independent samples. Statistical analyses were performed by using ordinary one-way analysis of variance (ANOVA) with Tukey's post hoc test. P values less than 0.05 were considered statistically significant differences among the compared groups.

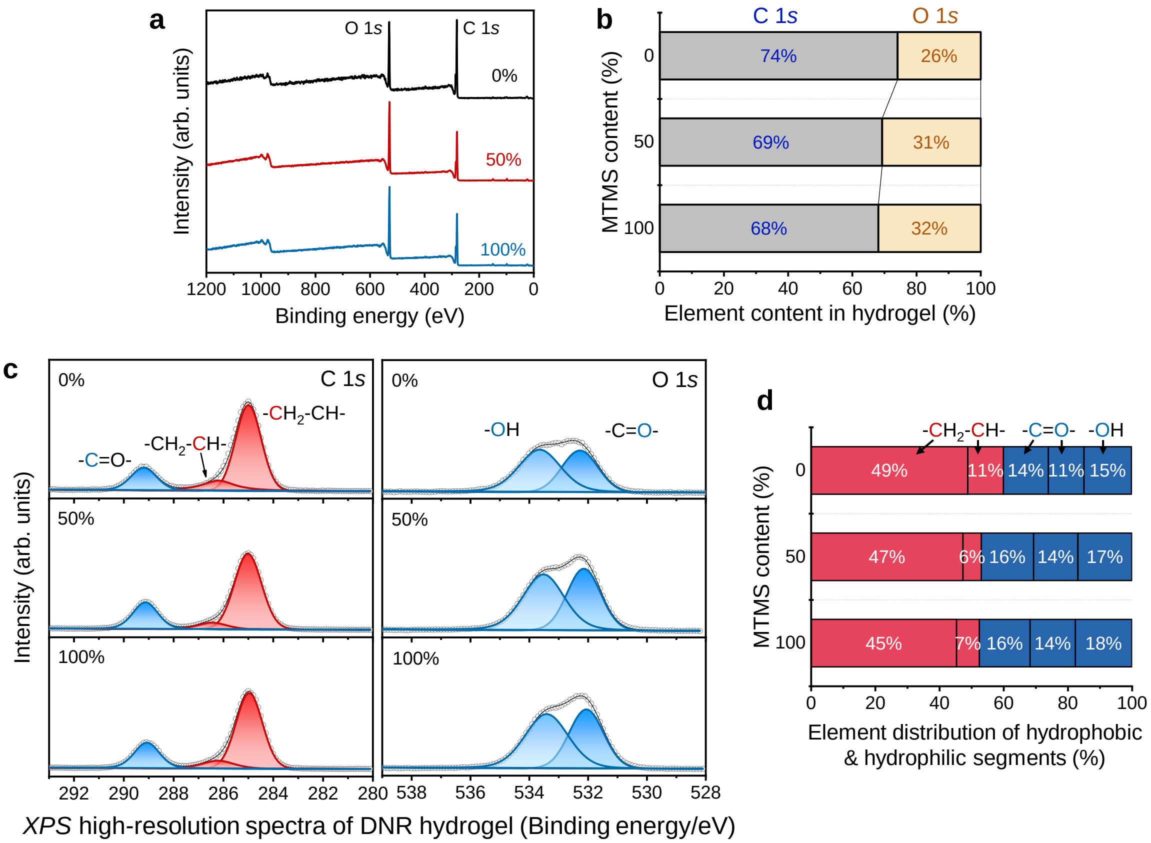

Supplementary Fig. 17 | a *XPS* spectra of DNR hydrogels prepared by the DNR molds with different crosslinking degrees of silicone chain. **b** Element contents of carbon and oxygen on the DNR hydrogel surface. The values were determined by integration of the C 1*s* and O 1*s* peak areas in **A**. **c** High-resolution *XPS* spectra of DNR hydrogels prepared by the DNR molds with different crosslinking degrees of silicone chain. **d** Statistics of the element content distribution of polymer segments in the DNR hydrogels.

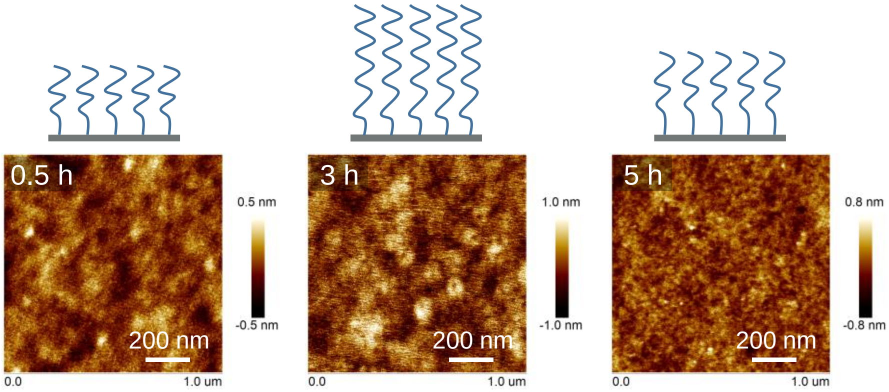

Supplementary Fig. 18 | AFM topographical images of the DNR mold surface grafted with different lengths of silicone chains, which was regulated by the condensation time of DMDMS (0.5 h, 3 h, and 5 h).

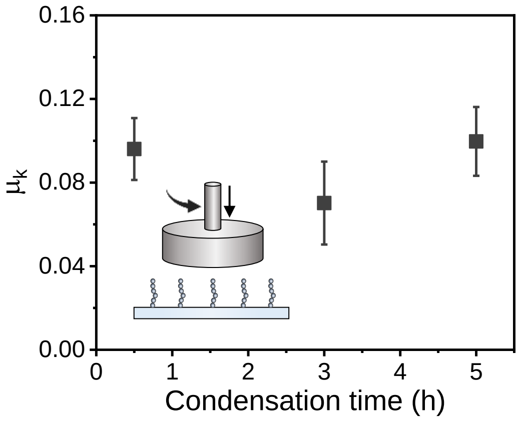

Supplementary Fig. 19 | Kinetic friction coefficient (μ_k) of the DNR mold surface grafted with different lengths of silicone chains. The chain length was determined by the condensation time of DMDMS, as shown in Fig. 2D. Mold surfaces with longer silicone chains demonstrate a lower μ_k, implying a higher flexibility and structural mobility of silicone chains. Data are shown as the mean ± SD; n = 3 independent samples.

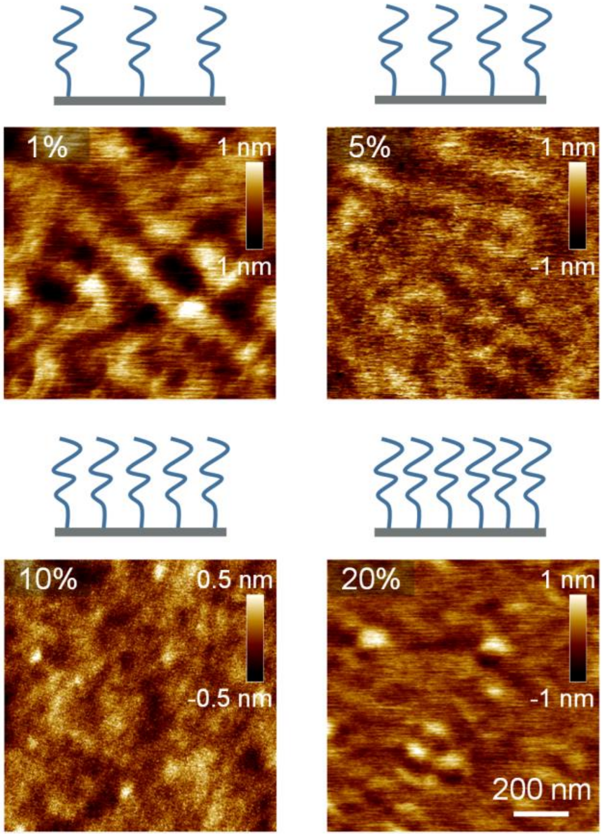

Supplementary Fig. 20 | AFM topographical images of the DNR mold surface. Silicone chains with different densities were grafted onto the DNR mold surface by using various concentrations of DMDMS (1 wt%, 5 wt%, 10 wt%, and 20 wt%).

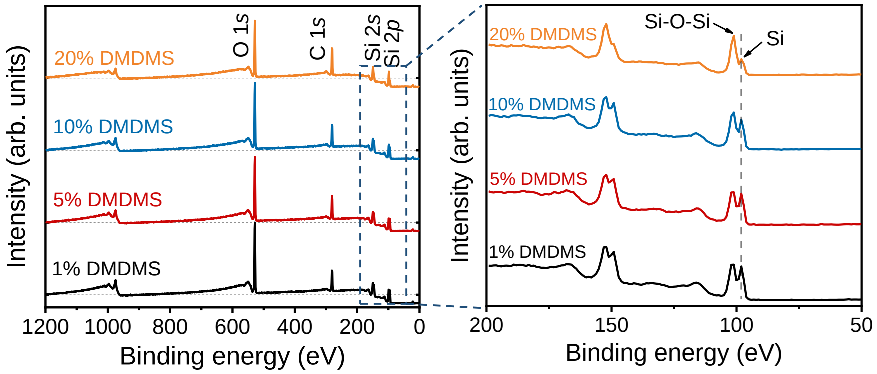

Supplementary Fig. 21 | *XPS* spectra of the DNR mold with different chain densities of silicone chains. The silicone chain was grafted onto a silicon wafer substrate, and the chain density was regulated by the DMDMS concentration (1 wt%, 5 wt%, 10 wt%, and 20 wt%).

Discussion: With higher DMDMS concentrations, a weaker signal assigned to silicon wafer (Si, 98.1 eV) can be observed for the DNR mold, suggesting a reduced substrate area and higher silicone chain density in the mold surface.

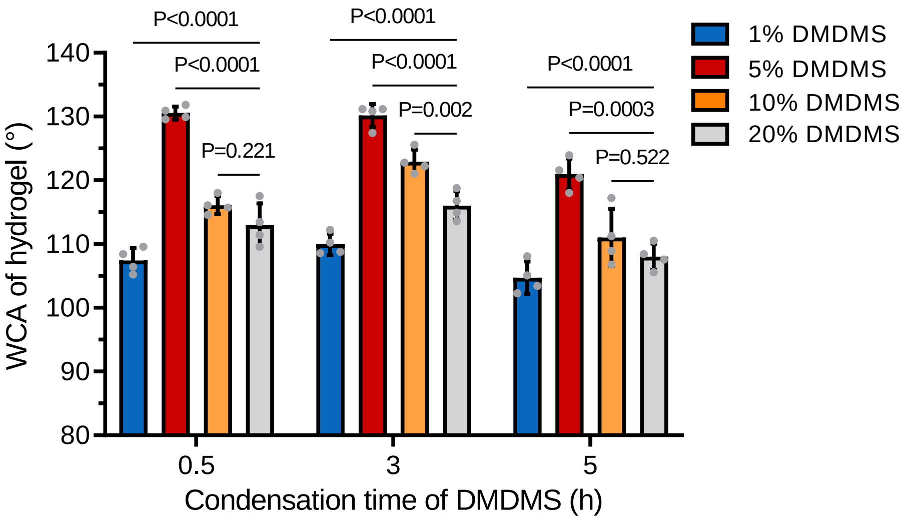

Supplementary Fig. 22 | WCA of PAA hydrogels prepared by DNR molds with different lengths and densities of grafted silicone chains. The length of silicone chains was regulated by different condensation time of DMDMS from 0.5 h to 5 h, where the density was regulated by various concentration of DMDMS from 1 wt% to 20 wt%. Data are shown as the mean ± SD; n = 4 independent samples. Statistical analyses were performed by using ordinary one-way analysis of variance (ANOVA) with Tukey's post hoc test. P values less than 0.05 were considered statistically significant differences among the compared groups.

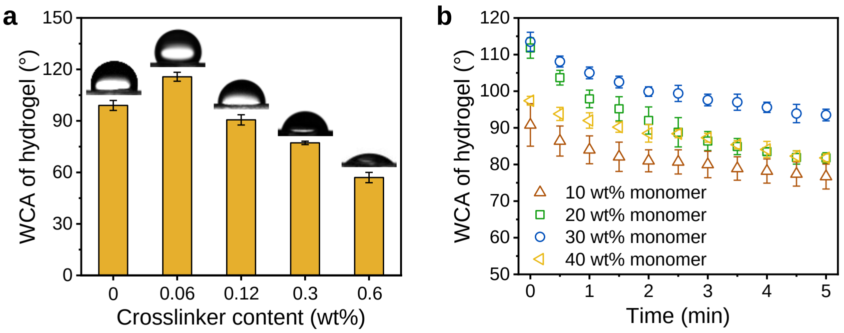

Supplementary Fig. 23 | Influence of hydrogel crosslink density and polymer content on hydrogel surface wettability regulated by the DNR strategy. a WCA of hydrogels prepared with different contents of the crosslinker MBAA (*N,N'*-methylene-bisacrylamide). Inset is corresponding WCA images. **b** WCA evolution of hydrogels prepared with different concentrations of monomer. PAA hydrogel was used as the model hydrogel. Data are shown as the mean ± SD; n = 4 independent samples.

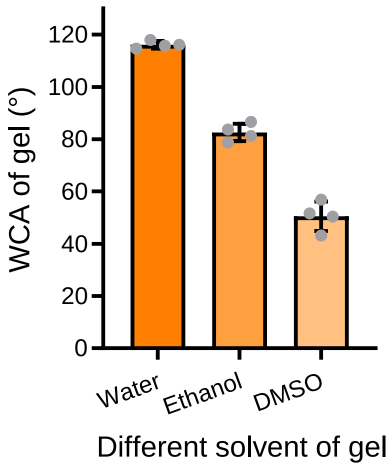

Supplementary Fig. 24 | WCA of PAA gels prepared using the DNR strategy with different polar solvents, including water, ethanol, and DMSO (dimethylsulfoxide). The preparation process of the gels with ethanol and DMSO is the same with the hydrogel except the change of solvent. Data are shown as the mean ± SD; n = 4 independent samples.

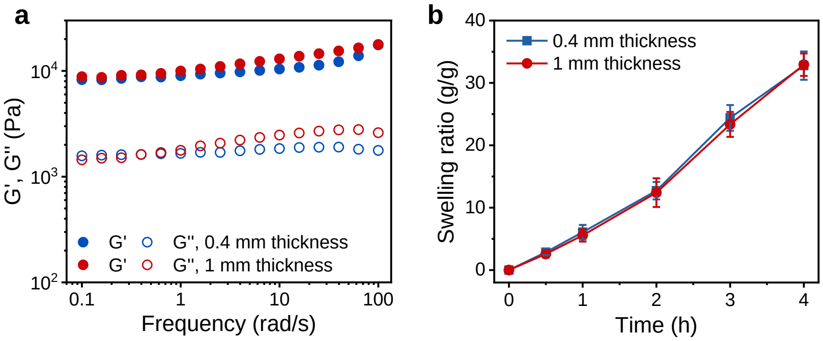

Supplementary Fig. 25 | a Rheological frequency sweep and **b** swelling ratio of DNR hydrogels with different thickness, including 0.4 mm and 1 mm. Values in **b** are shown as the mean ± SD; n = 3 independent samples.

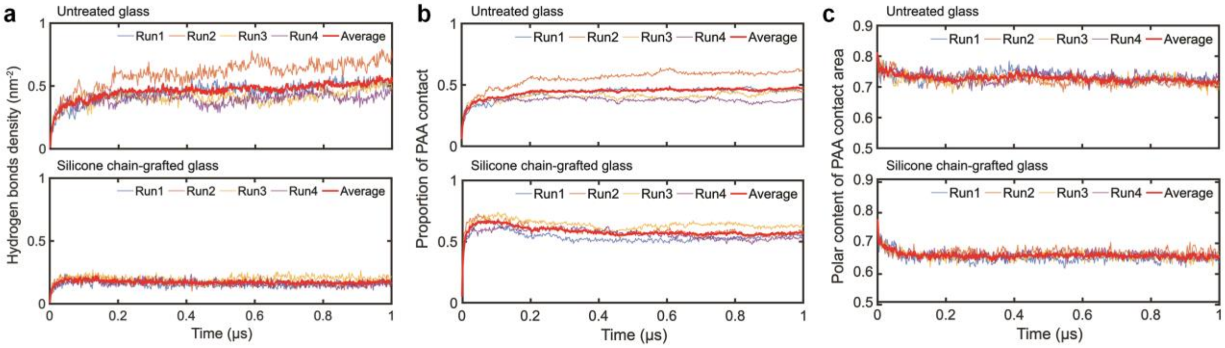

Supplementary Fig. 26 | Time series plots for the MD simulations of PAA hydrogel formation on untreated glass and silicone chain-grafted glass. a Density of interfacial hydrogen bonds formed between the PAA hydrogel and molds. **b** Proportion of the mold surface area in contact with the PAA hydrogel surface network. **c** Polar contents in the surface network of PAA hydrogels contacting the different mold surfaces.

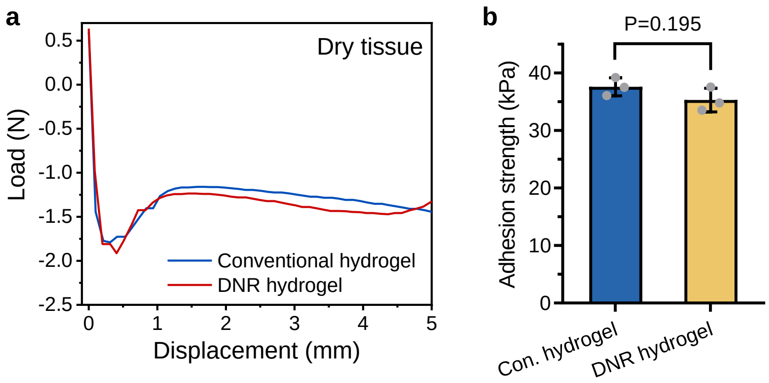

Supplementary Fig. 27 | a Force curves of the pull-off test on dry tissue surface. Conventional and DNR hydrogels based on PAA were applied to dry porcine skin for the tests. **b** Adhesion strength of conventional (abbreviated as Con.) and DNR hydrogels on dry porcine skins, which was calculated by dividing peak pull-off forces by the contact area between hydrogels and tissue. Values in **b** are shown as the mean ± SD; n = 3 independent samples. Statistical analysis was performed by using two-tailed Student's *t* test. P values less than 0.05 were considered statistically significant differences among the compared groups.

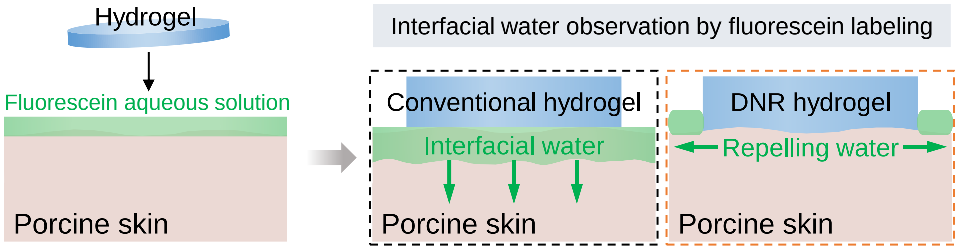

Supplementary Fig. 28 | Schematic illustration of the effective repelling of fluorescently labeled interfacial water by the DNR hydrogel. We verified the repelling of interfacial water by the DNR hydrogel on porcine skin by observing a fluorescein aqueous solution added to the hydrogel–tissue interface by a fluorescence microscope.

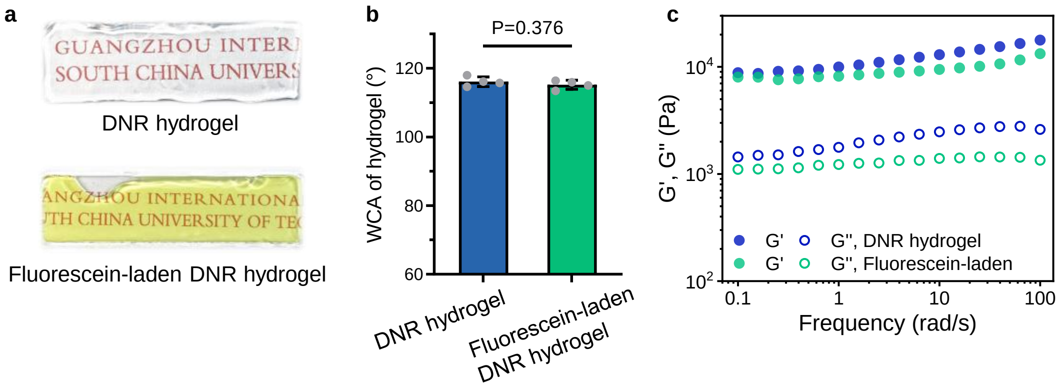

Supplementary Fig. 29 | Comparison of the DNR hydrogel before and after loading of sodium fluorescein molecules. a Photo images, **b** WCA, and **c** rheological frequency sweep of DNR hydrogel and fluorescein-laden DNR hydrogel. Values in **b** are shown as the mean ± SD; n = 4 independent samples. Statistical analysis was performed by using two-tailed Student's *t* test. P values less than 0.05 were considered statistically significant differences among the compared groups.

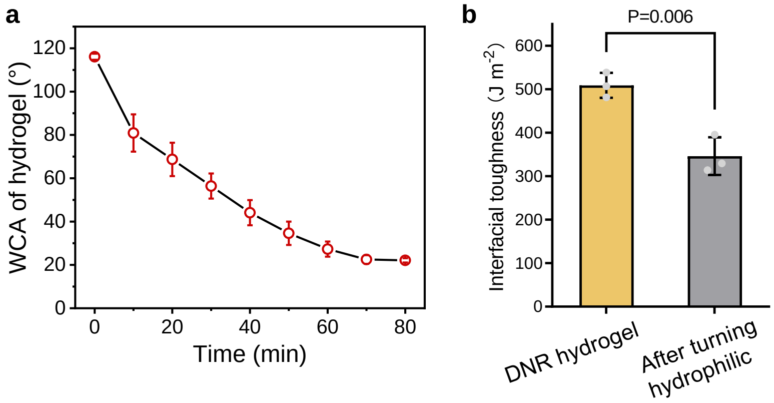

Supplementary Fig. 30 | a WCA evolution of the DNR hydrogel in 80 min, which shows the hydrophobicity to hydrophilicity transition of DNR hydrogel over time. **b** Adhesion toughness of DNR hydrogel before and after turning hydrophilic adhered on wet porcine skin. Data are shown as the mean ± SD; n = 3 independent samples. Statistical analysis was performed by using two-tailed Student's *t* test. P values less than 0.05 were considered statistically significant differences among the compared groups.

Discussion: The WCA evolution profile of DNR hydrogel in 80 min shows the full transition of the hydrogel surface wettability from hydrophobicity to hydrophilicity over time. This wettability transition may weaken the adhesion of DNR hydrogel to wet tissues to some extent. Nevertheless, the early hydrophobicity of DNR hydrogel facilitates the bioadhesion on wet tissues.

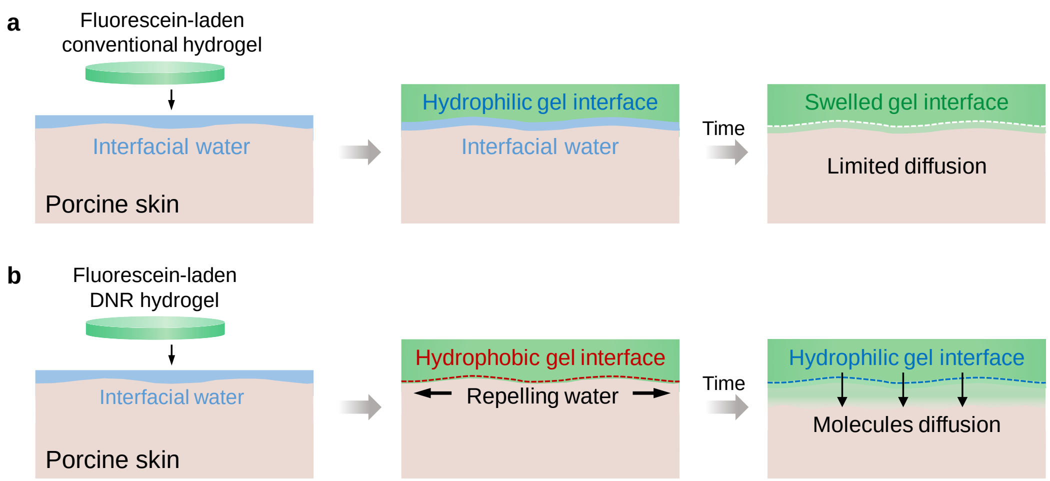

Supplementary Fig. 31 | Schematic illustration of the transdermal delivery of fluorescein by **a** conventional and **b** DNR hydrogels to porcine skin tissue.

Discussion: Due to the initial surface hydrophobicity, DNR hydrogels can repel interfacial water at the beginning and then promote the diffusion of fluorescein molecules into skin tissue as the hydrogel surface gradually turned hydrophilic over time. In contrast, conventional hydrogels would be swollen by the interfacial water and the diffusion of fluorescein molecules was hindered by the swollen gel interface.

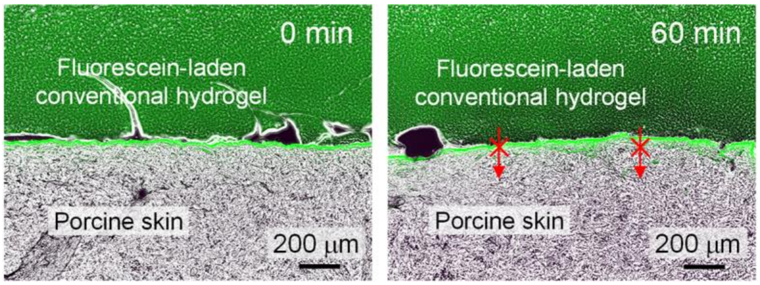

Supplementary Fig. 32 | The merge of bright field and fluorescence images of cryosectioned porcine skins demonstrating the hindered transdermal delivery of fluorescein into skin tissue by the conventional hydrogel.

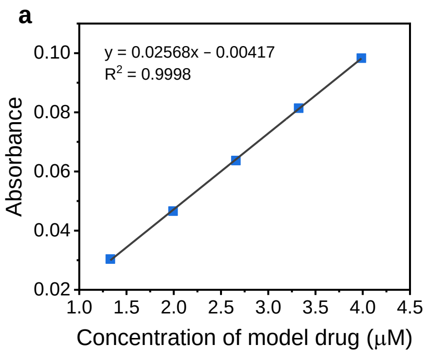

Supplementary Fig. 33 | a The standard curve of sodium fluorescein, which was determined by a UV–vis spectrophotometer at 474 nm. **b** The cumulative release of sodium fluorescein from the DNR hydrogel. Values in **b** are shown as the mean ± SD; n = 3 independent samples.

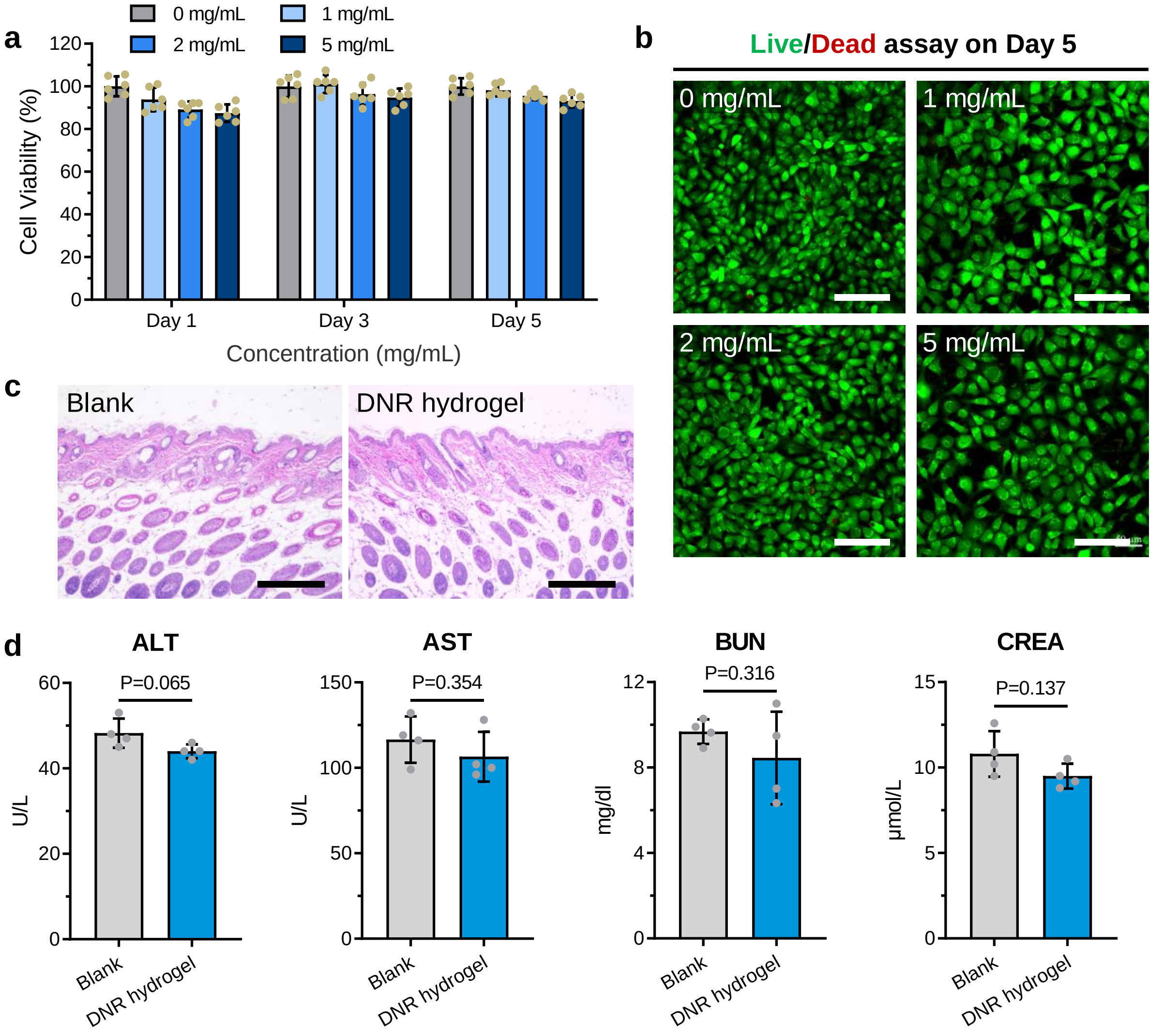

Supplementary Fig. 34 | Evaluation of the biocompatibility of DNR hydrogel. a Cytotoxicity of DNR hydrogel by MTT method. The L929 cells were cultured in DMEM with different concentration of hydrogel extracts for different periods. Control group: 0 mg/mL; DNR hydrogel group: 1–5 mg/mL. **b** Representative images of L929 cells with live/dead staining after incubation with different concentration of hydrogel extracts for 5 days (scale bar: 100 μm; control group: 0 mg/mL; DNR hydrogel group: 1–5 mg/mL). **c** Hematoxylin-eosin (H&E) staining images of skin tissue after subcutaneous embedding of DNR hydrogel in Balb/C mouse skin incision model for 14 days (N = 4 biologically independent samples in each group; scale bar: 300 μm). **d** Blood biochemistry analysis of liver and kidney function markers (ALT, AST, BUN, and CREA). Liver function assessment: ALT (alanine aminotransferase), AST (aspartate aminotransferase), BUN (blood urea nitrogen). Kidney function assessment: CREA (creatinine). Values in **a** are presented as the mean ± SD, n = 6 independent samples; values in **d** are presented as the mean ± SD, n = 4 independent samples. Statistical analyses were performed by using two-tailed Student's t test. P

values less than 0.05 were considered statistically significant differences among the compared groups.
