# Supplementary material for: Surface hydrophobization of hydrogels via interface dynamics-induced network reconfiguration
Source: Nat Commun. 2024 Jan 3;15:239. doi: 10.1038/s41467-023-44646-5 (PMC10764767; doi:10.1038/s41467-023-44646-5)
Supplement: Supplementary file 3 — Description of Additional Supplementary Files [file 41467_2023_44646_MOESM3_ESM.pdf]

Description of Additional Supplementary Files

File name: Supplementary Movie 1
Description: A 5-μL water drop on PAA-based DNR hydrogel. The play speed is 20×.

File name: Supplementary Movie 2
Description: A 5-μL water drop on PAA hydrogel prepared by silicone oil-coated mold. The play speed is 20×.

File name: Supplementary Movie 3
Description: MD simulation visualizations of a single PAA chain diffusing on the silicone chain-grafted mold surface with 0%/50%/100% silicone chains restrained.

File name: Supplementary Movie 4
Description: Tough bioadhesion of PAA-based DNR hydrogel on a wet porcine heart.

File name: Supplementary Movie 5

Description: Adhesion of PAA-based conventional hydrogel on a wet porcine heart.
