# Supplementary material for: Surface hydrophobization of hydrogels via interface dynamics-induced network reconfiguration
Source: Nat Commun. 2024 Jan 3;15:239. doi: 10.1038/s41467-023-44646-5 (PMC10764767; doi:10.1038/s41467-023-44646-5)
Supplement: Supplementary file 10 — Source data [file 41467_2023_44646_MOESM10_ESM.zip › Source data/Figure S9/Roughness_glass.pdf]

Product (Produkt)	Mat.No. (Mat.Nr.)	Ser.No. (Serien-Nr.)

Customer (Kunde)	Order (Auftrag)
	**6012**
Test Standard /Ser.No. (Prüfnormal /Serien-Nr.)	Probe arm / Ser.No. (Tastarm / Serien-Nr.)
**Prüftaster TP-TKU400 / 133431**	**TAM1**
Operator (Prüfer)	measuring direction
**T. Eichenmüller**	**bottom longitudinal**

JENOPTIK

1-1

Ra 2.386 µm

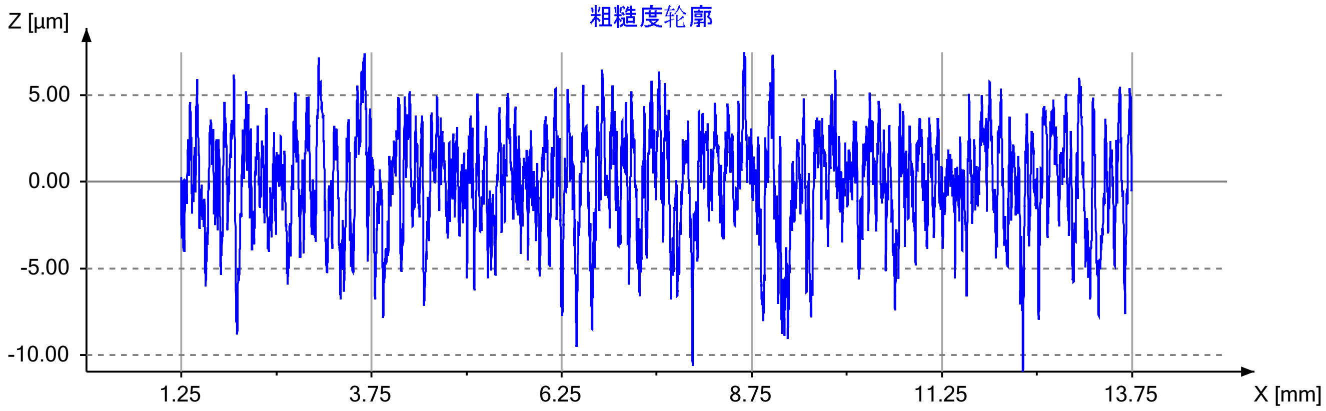

评价条件		对齐:	开	测量范围:	400 µm
λs:	关	lp:	14.995 mm	测量方向:	从左
λc:	2.50 mm	lr:	2.500 mm	测量点:	30000
λf:	关	lw:	2.500 mm	测量点间距:	0.5 µm
删除形状:	关	线性测量点 Xz4		探针半径:	0.002 mm
滤波 ISO 4287:	ISO 16610-21	探测距离:	15.00 mm	测头类型:	TKU400
滤波 ISO 135…	---	速度:	0.50 mm/s	附加参数	

1-2

Ra 2.370 µm

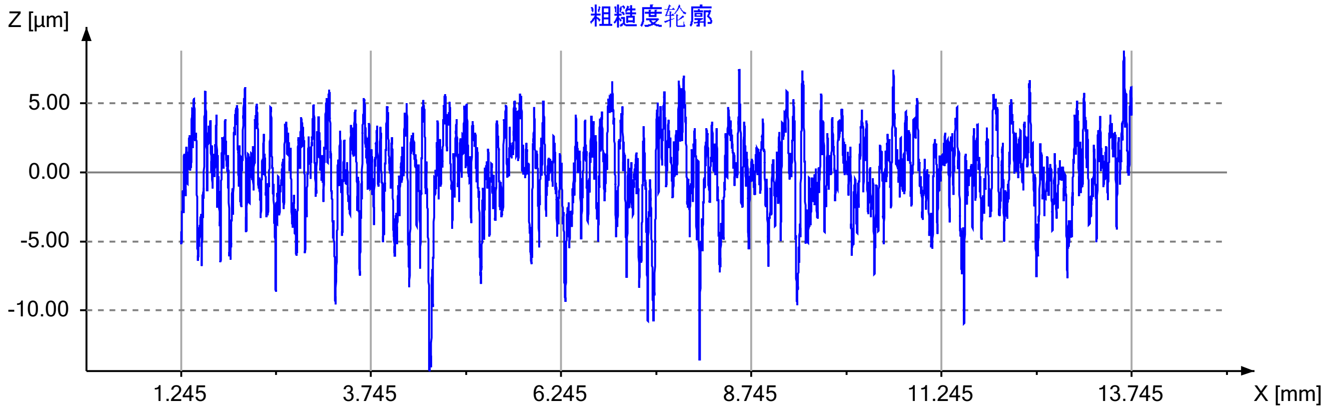

评价条件		对齐:	开	测量范围:	400 µm
λs:	关	lp:	14.995 mm	测量方向:	从左
λc:	2.50 mm	lr:	2.500 mm	测量点:	30000
λf:	关	lw:	2.500 mm	测量点间距:	0.5 µm
删除形状:	关	线性测量点 Xz5		探针半径:	0.002 mm
滤波 ISO 4287:	ISO 16610-21	探测距离:	15.00 mm	测头类型:	TKU400
滤波 ISO 135…	---	速度:	0.50 mm/s	附加参数	

Product (Produkt)	Mat.No. (Mat.Nr.)	Ser.No. (Serien-Nr.)

Customer (Kunde)	Order (Auftrag)
	**6012**
Test Standard /Ser.No. (Prüfnormal /Serien-Nr.)	Probe arm / Ser.No. (Tastarm / Serien-Nr.)
**Prüftaster TP-TKU400 / 133431**	**TAM1**
Operator (Prüfer)	measuring direction
**T. Eichenmüller**	**bottom longitudinal**

JENOPTIK

1-3

Ra	2.236 µm

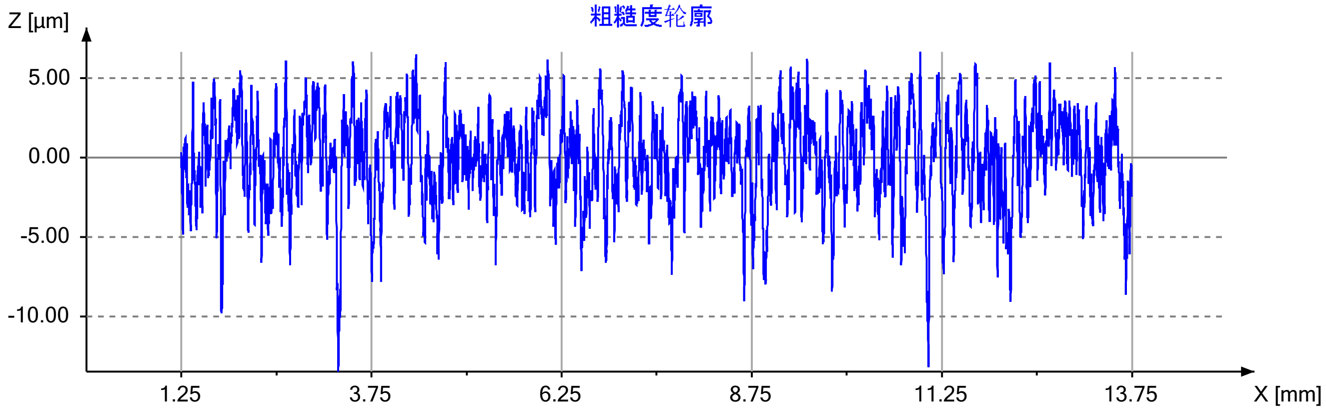

评价条件		对齐:	开	测量范围:	400 µm
λs:	关	lp:	14.996 mm	测量方向:	从左
λc:	2.50 mm	lr:	2.500 mm	测量点:	30000
λf:	关	lw:	2.500 mm	测量点间距:	0.5 µm
删除形状:	关	线性测量点 Xz6		探针半径:	0.002 mm
滤波 ISO 4287:	ISO 16610-21	探测距离:	15.00 mm	测头类型:	TKU400
滤波 ISO 135…	---	速度:	0.50 mm/s	附加参数	
